# Unexpected recovery from longterm renal failure in severe diffuse proliferative lupus nephritis

**DOI:** 10.1186/1471-2369-13-81

**Published:** 2012-08-06

**Authors:** Sophia Ross, Kerstin Benz, Katja Sauerstein, Kerstin Amann, Jörg Dötsch, Katalin Dittrich

**Affiliations:** 1 Department of Pediatric Nephrology, University of Erlangen-Nürnberg, Loschgestr. 15, Erlangen, D-91054, Germany; 2 Department of Nephropathology, University of Erlangen-Nürnberg, Erlangen-Nürnberg, Germany; 3 Department of Pediatrics, University of Köln, Köln, Germany; 4 Hospital for Children and Adolescents, University of Leipzig, Leipzig, Germany

**Keywords:** Proliferative lupus nephritis, Acute renal failure, Immunosuppressive treatment, Mycophenolate mofetil, Remission

## Abstract

**Background:**

Severe renal manifestation of systemic lupus erythematosus (SLE) is not uncommon and is associated with an indeterminate prognosis. Complete remission can be obtained, however, at least in the young when chronic lesions are absent and adequate anti-inflammatory therapy is immediately initiated.

**Case presentation:**

We report the unusual case of a 12-year-old girl who presented with severe oliguric renal failure, macrohematuria and skin rash. Renal biopsy revealed the diagnosis of severe diffuse proliferative glomerulonephritis (GN) with cellular crescents in 15 out of 18 glomeruli and full-house pattern in immunofluorescence indicating lupus nephritis IVB according to WHO, IV-G(A) according to ISN/RPS classification. The serological parameters confirmed the diagnosis of SLE and the patient was immediately treated with methylprednisolone, cyclophosphamide and immunoadsorption. Initially, despite rapid amelioration of her general condition, no substantial improvement of renal function could be achieved and the patient needed hemodialysis treatment for 12 weeks. Unexpectedly, in the further follow-up at first diuresis increased and thereafter also creatinine levels substantially declined so that hemodialysis could be discontinued. Today, 6 years after the initial presentation, the patient has normal renal function and a SLEDAI score of 0 under a continuous immunosuppressive therapy with Mycophenolate mofetil (MMF) and low dose steroid.

**Conclusion:**

Despite the severity of the initial renal injury and the unfavourable renal prognosis the kidney apparently has a tremendous capacity to recover in young patients when the damage is acute and adequate anti-inflammatory therapy is initiated without delay.

## Background

Lupus nephritis is a serious complication of systemic lupus erythematosus (SLE). Especially in active diffuse proliferative lupus nephritis (WHO class IV, ISN/RPS IV A) a poor outcome with a high risk of end stage renal disease has been reported [1]. A significant correlation between proteinuria, GFR and severity of nephritis has been observed. Proliferative lupus nephritis features the most severe proteinuria and the lowest GFR-values [2]. Despite early and thoroughly elaborated aggressive immunosuppressive therapy many of these patients are faced with end stage renal disease and continuous dialysis.

We report the case of a 12-year-old girl with severe diffuse proliferative lupus nephritis type IV with cellular crescents in 80% of glomeruli and oligoanuria at presentation requiring dialysis treatment for several months. However, due to intensive immunosuppressive treatment complete recovery of renal function could finally be achieved.

## Case report

A 12-year-old Caucasian girl presented at her general practitioner because of swollen legs and spotted skin macular rash over her face, on her arms and on her chest. She was also complaining about headaches, abdominal pain, arthralgia and macroscopic haematuria. In the last few weeks she had gained 4 kg body weight. At admission to the children’s hospital she presented in a poor general state with hypertension of 143/99 mmHg, considerable oedema of the legs and facial rash. Laboratory results showed anemia (hemoglobin 9.2 g/dl), leukopenia (2.650/μl), normal thrombocyte counts, acute renal failure (creatinine 5.8 mg/dl, urea 166 mg/dl) with hyperkalemia (6.4 mmol/l) and metabolic acidosis. Furthermore, macroscopic haematuria with dysmorphic erythrocytes, proteinuria of 6.6 g/m^2^ body surface area per day, serum albumin of 19.6 g/l and oliguria were observed. Abdominal ultrasound demonstrated ascites but no pleural or pericardial effusion, enlarged kidneys with increased echogenicity and lacking corticomedullary differentiation.

Further diagnostic tests showed extremely low complement levels (C3 and C4 below detection level), markedly elevated anti-nuclear-antibodies (ANA 1:320; normal value <1:100) and a particularily strong increase of anti-double-stranded nuclear antibodies (anti-dsDNA, 1500 U/ml; normal value <7 U/ml). Other autoimmune phenomena were carefully excluded: tests for ANCA, anti-GBM, anti-phospholipid antibodies, and anti-extractable nuclear antigen all yielded negative results. Neither the patient nor her relatives had any other immune abnormality at present or in the past history. The patient did not show any signs of hemolysis or abnormal coagulation.

**Figure 1:**
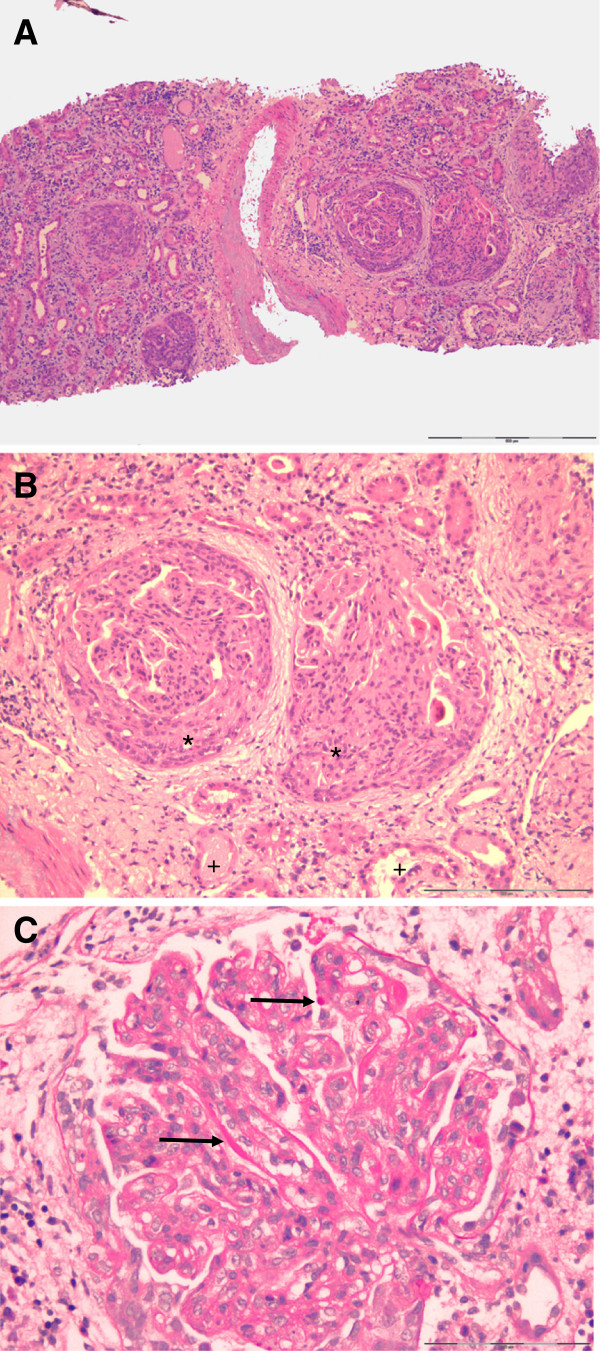
**Renal biopsy.A**, **B**, **C**: Active diffuse proliferative lupus glomerulonephritis with cellular crescents (*) in 15 of 18 glomeruli, marked inflammation of the interstitium and signs of acute tubular necrosis (+). Please note that there is no chronic damage in the kidney. In Figure 1C also markedly thickened, stiff appearing glomerular basement membranes due to intramembranous and subepithelial immune deposits (arrow) are seen. Light microscopy, PAS Stain. Magnifications x 10 (A), x 20(B), x 40(C).

**Figure 2:**
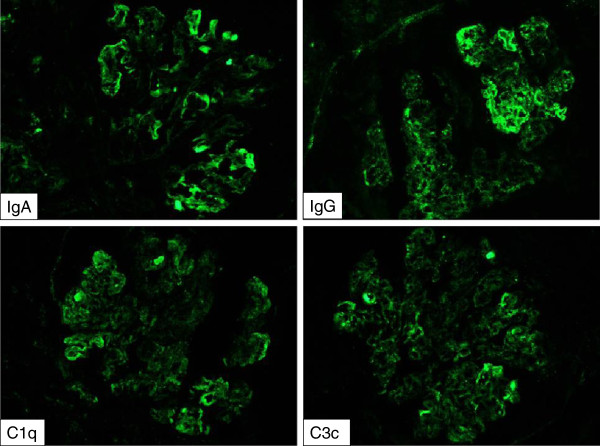
**Immunofluorescence of the kidney biopsy.** Here, the typical “full-house” pattern with intense (+++) granular staining for IgA, IgG, IgM (not shown), C1 and C3 in a diffuse mesangiocapillary pattern can be seen.

Renal biopsy performed immediately in the next morning confirmed the diagnosis of renal involvement in lupus erythematosus showing diffuse proliferative glomerulonephritis (GN) with cellular crescents in 15 of 18 glomeruli, severe interstitial oedema, acute inflammation and acute tubular necrosis (Figure 1a-c). On immunofluorescence, IgA, IgG, IgM as well as C1q and C3c depositions, i.e. a so-called fullhouse pattern, were detected in a mesangiocapillary pattern (Figure 2). This pattern of immune complex deposition was also confirmed on electron microscopy. Of note, tubuloreticular or fingerprint like structures were not present. Therefore, a very active diffuse global proliferative lupus nephritis (WHO IV B, ISN/RPS IV-G (A)) was diagnosed. Of note, no chronic changes, i.e. tubular atrophy or interstitial fibrosis, were seen in the kidney. Due to mild depressive mental state an additional EEG was performed. The result showed dysrhythmic oscillations that led us to hypothesize cerebral vasculitis. MRI angiography, however, was normal.

**Figure 3:**
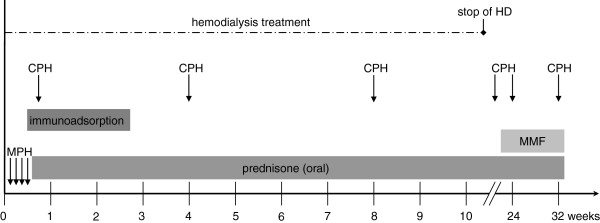
**Immunosuppressive treatment regime: The patient was initially treated with 4 methylprednisolone pulses (MPH), 6 cyclophosphamide pulses (CPH) and immunoadsorption (10 courses).** Mycophenolate mofetil (MMF) was started as maintenance therapy 3 months after admission. After 12 weeks permanent discontinuation of hemodialysis treatment was successful.

After the diagnosis of severe proliferative lupus nephritis was confrmed by renal biopsy we intensively discussed the therapeutic options and decided to immediately initiate the standard immunosuppressive treatment schedule according to the Euro Lupus Trial [3] with methylprednisolone pulse therapy (4 pulses with 400/200/200/100 mg/m² BSA respectively) followed by oral prednisone administration tapered from 60 to 10 mg/m²/48 h over an 48 week period, intravenous cyclophosphamide pulse (in total 6 pulses in 4 weeks interval) and additionally 10 courses of immunoadsorption over a period of 2 weeks. The general clinical condition rapidly improved, but the patient still needed intermittent hemodialysis due to oligoanuria and antihypertensive treatment with amlodipine, atenolol and enalapril. She received pneumocyctis carinii prophylaxis and standard therapy for end stage renal disease. As no substantial improvement of renal function could be achieved after the 2^nd^ CPH pulse and immunoadsorption, the patient received a Cimino fistula for intermittent hemodialysis. Unexpectedly, in the further follow-up diuresis increased and subsequently also serum creatinine levels substantially improved so that hemodialysis could be discontinued about 12 weeks after initial admission. Maintenance immunosuppressive therapy with mycophenolate mofetil (MMF) was started 3 months after admission (Figure 3). Of course at this stage a re-biopsy would have been very helpful to adjust the therapy and indeed this was discussed with the patient and her parents who refused in view of the positive clinical course and the well-known, albeit low risk of a kidney biopsy.

At further follow-up creatinine levels, proteinuria and complement C3 values improved and returned to normal range. ANA and dsDNA antibodies were only intermittently detectable. The patient had 2 short episodes of clinical relapse with leukopenia, proteinuria and increase in dsDNA that were successfully treated for 4 weeks with an increased dosage of oral steroids. Today, six years after the initial presentation the patient is in complete remission under permanent immunosuppression with MMF (2x750mg) in combination with prednisone (5 mg/48 h) and enalapril (40 mg). She has normal renal function with creatinine levels continuously in the normal range, no proteinuria and no other signs of SLE.

## Discussion

The most striking observation in the presented case is the unexpected complete recovery of renal function after a long period of dependency on hemodialysis in a 12 year old girl with severe proliferating lupus nephritis (WHO class IV) and 80% glomeruli with cellular crescents.

In ANCA associated pauci-immune glomerulonephritis it is known that a high number of crescents correlates with the potential of recovery and that amelioration by immunosuppressive therapy can be achieved [4]. In proliferative lupus nephritis, however, a high activity index with more than 50% of glomerular crescents is a risk factor associated with further progression of nephritis [5]. In our patient kidney function severely deteriorated and oliguria persisted despite rapid improvement of general condition after steroid and cyclophosphamide treatment in combination with immunoadsorption. Due to the known bad prognosis of proliferative lupus nephritis [6,7] and in view of the young age of the patient, we decided to apply an aggressive immunosuppressive treatment to try to rescue the patient from end stage renal failure. Although data on the efficacy of immunoadsorption and plasmapheresis in SLE are controversial, both are rescue therapies in patients with various autoimmune diseases. Immunoadsorption is associated with an acceptable risk of bacterial and viral infection and it has been recently shown that it leads to reduction in proteinuria, anti-dsDNA antibodies and general disease activity in SLE [8].

After the 2^nd^ CPH pulse and 5 weeks after admission the reasonability of further CPH pulses was debated as the patients general condition was good. Serological parameters were back to normal and improvement of kidney function seemed unlikely. The worth and risk of the treatment was discussed in view of the well-known severe side effects of an aggressive immunosuppression i.e. infections, hypertension, nonvascular bone necrosis, osteoporosis but also lethal outcome are known [9]. A decision was made for the continuation of the treatment and fortunately no systemic infection, no bone affections or comparable severe side effects occurred. Interestingly and unexpected in view of the very grave histological findings at presentation and the long dependency on dialysis treatment, her renal function recovered so that hemodialysis could be stopped after 12 weeks. Under continuous MMF and low dose steroid therapy renal function remained stable for now 6 years and the patient is in a very good general condition with normal renal function and without proteinuria. Only 2 short episodes of clinical relapses of SLE occurred that were successfully treated with increased steroid dosage for a 4 week period. Although complete remission of proliferative glomerular lesions is very rare one could imagine that the reparative capacity of the young and adolescent kidney in the absence of any chronic glomerular or tubulointerstitial lesions is high enough for nearly complete recovery from renal lesions. We have seen a similar favourable course in a 16 year old patient with acute renal failure due to severe crescentic anti-GBM GN, who recovered after more than one year of chronic hemodialysis treatment [10] and also Benseler et al. [11] described some pediatric patients with lupus nephritis in whom dialysis could be stopped after adequate treatment.

The issue of renal recovery is certainly complex: it is well known that severe lupus nephritis can result in a need for dialysis in SLE patients, many of whom recover despite being on prolonged dialysis. As shown by Chu and Folkert [12] recovery of renal function in chronic dialysis patients occurred mainly in cases with autoimmune disease, especially lupus nephritis. The rate of renal function recovery in this group of patients approaches 10–28%, and occurs at a median time of 3–18 months [13-17]. Hence the treatment of severe class IV lupus nephritis should be continued for up to 6 months in order to promote recovery as was the case in our patient.

## Conclusion

In the absence of chronic lesions of the kidney and probably partly due to the high capacity of the kidney in children and young adults to recover, complete remission of renal failure caused by severe proliferative lupus nephritis even after several months of hemodialysis is possible when an aggressive anti-inflammatory and immunosuppressive treatment regime according to standard protocols is used without delay. The favourable course of the case presented here may encourage physicians confronted with similar cases. In elderly patients with comparable renal disease mostly chronic lesions are already present so that a similar positive outcome cannot necessarily be expected.

## Consent

Written informed consent was obtained from the patient for publication of this Case report and any accompanying images. A copy of the written consent is available for review by the Series Editor of this journal.

## Competing interests

The authors declare that they have no competing interests.

## Authors’ contributions

SR, KS, KB and KA drafted the manuscript. KB, KA, JD and KD conceived of the study, and participated in its design and coordination and helped to draft the manuscript. All authors read and approved the final manuscript.

## Pre-publication history

The pre-publication history for this paper can be accessed here:

http://www.biomedcentral.com/1471-2369/13/81/prepub
